# Compositional variation of the human fecal microbiome in relation to azo-reducing activity: a pilot study

**DOI:** 10.1186/s13099-021-00454-0

**Published:** 2021-10-08

**Authors:** Sara A. Zahran, Marwa Ali-Tammam, Amal E. Ali, Ramy K. Aziz

**Affiliations:** 1 Department of Microbiology and Immunology, Faculty of Pharmacy, Future University in Egypt, New Cairo, 11835 Egypt; 2 Department of Microbiology and Immunology, Faculty of Pharmacy, Cairo University, Cairo, 11562 Egypt; 3 The Center for Genome and Microbiome Research, Faculty of Pharmacy, Cairo University, Cairo, 11562 Egypt; 4 Microbiology and Immunology Research Program, Children’s Cancer Hospital Egypt 57357, Cairo, 11617 Egypt

**Keywords:** Azoreductase, Gut microbiota, Firmicutes, Bacteroidetes, 16S rRNA, Pharmacomicrobiomics

## Abstract

**Background:**

Through an arsenal of microbial enzymes, the gut microbiota considerably contributes to human metabolic processes, affecting nutrients, drugs, and environmental poisons. Azoreductases are a predominant group of microbiota-derived enzymes involved in xenobiotic metabolism and drug activation, but little is known about how compositional changes in the gut microbiota correlate with its azo-reducing activity.

**Results:**

To this end, we used high-throughput 16S rRNA amplicon sequencing, with Illumina MiSeq, to determine the microbial community composition of stool samples from 16 adults with different azo-reducing activity. High azo-reducing activity positively correlated with the relative abundance of phylum Firmicutes (especially genera *Streptococcus* and *Coprococcus*) but negatively with phylum Bacteroidetes (especially genus *Bacteroides*). Typical variations in the Firmicutes-to-Bacteroidetes and *Prevotella*-to-*Bacteroides* ratios were observed among samples. Multivariate analysis of the relative abundance of key microbial taxa and other diversity parameters confirmed the Firmicutes proportion as a major variable differentiating high and non-azo-reducers, while Bacteroidetes relative abundance was correlated with azo-reduction, sex, and BMI.

**Conclusions:**

This pilot study showed that stool samples with higher azo-reducing activity were enriched in Firmicutes but with relatively fewer Bacteroidetes. More samples and studies from different geographical areas are needed to bolster this conclusion. Better characterization of different azoreductase-producing gut microbes will increase our knowledge about the fate and differential human responses to azodye-containing drugs or orally consumed chemicals, thus contributing to efforts towards implementing microbiome testing in precision medicine and toxicology.

**Supplementary Information:**

The online version contains supplementary material available at 10.1186/s13099-021-00454-0.

## Background

The human gastrointestinal tract is a large interface between the host, environmental factors, and antigens in the human body. The gut microbiota represents ten times the number of nucleated human cells and harbors two orders of magnitude more genomic content than the human genome [1]. Members of the gut microbiota form a complex, mutually beneficial relationship, which substantially contributes to human metabolic processes via their extended gene pool and their encoded enzymes [2, 3]. Among the most predominant enzymes expressed by several members of the human gut microbiota are azoreductases, which catalyze the reduction of azo-bonds, activating pharmaceutical dosage forms or degrading food additives [4–7].

Among azo compounds whose reduction is largely mediated by the gut microbiota are (i) azo-antibacterial pro-drugs based on sulfanilamide (e.g., prontosil and neoprontosil), (ii) a range of 5-aminosalicylic acid pro-drugs used in the treatment of ulcerative colitis and inflammatory bowel conditions [8, 9], and (iii) drug-delivery systems that target the colon depending on the azoreductase enzymes produced by the large intestinal microbiota [6]. Thus, the metabolism and bioavailability of such drugs are largely affected by the azo-reducing capability of the gut microbiota, and compositional changes in the gut microbial community lead to differential human responses toward these drugs. Personalized therapeutics, classically based on an individual’s genetics, is being expanded to the association between the microbiome and bioavailability, treatment outcome, and toxicity of a given drug. Pharmacomicrobiomics [5, 10] and toxicomicrobiomics [11, 12], as subfields of precision medicine, are becoming necessary for developing new preventive and therapeutic strategies [13, 14]. One of the most attractive enzymes for pharmacomicrobiomic studies is the group of azoreductases [4, 7].

Several researchers have isolated, purified, and biochemically characterized different azoreductases from aerobic and anaerobic microorganisms, some of which are members of the human gut microbiota. They identified their encoding genes, and described their catalytic activity, cofactor requirement, and biophysical characteristics (e.g., [15–21]). However, less attention was given to define the relative abundance of azoreductase-producing microbes within the human gut and to relate compositional variations in the gut microbiota to their azo-reducing activity. Accordingly, this study aimed to explore the composition of the fecal microbiomes of a group of adults with no diagnosed diseases and try to relate their microbial community composition to their azo-reducing activity.

## Results

### Total decolorization activity of stool samples

Out of 16 collected stool specimens, six did not show any significant reduction in Brilliant Black level during the experiment time (up to 10 h), and are classified hereafter as the “grade zero” decolorization group. Two other specimens had moderate azo-reducing activity (only caused partial decolorization during the 10 h of the experiment), and their decolorization potential was classified as low grade or “grade one” (Additional file 1: Figure S1). The last eight specimens had higher azo-reducing activity (caused complete, or near complete, decolorization within the experiment time) and were assigned to a “grade two” category (Table 1 and Additional file 1: Figure S1).

**Table 1:** Azo-reducing activity of fresh stool samples against 0.06 M Brilliant Black

Sample number	Azo-reducing activity grade	Time to full decolorization (h)	% Decolorization at the end of the experiment (10 h) expressed as mean (± SD)%
S9	Grade zero (no degradation)	> 10	0 (± 1.8)%
S4		> 10	0 (± 0.8)%
S15		> 10	0 (± 1.4)%
S5		> 10	2.7 (± 1.2)%
S12		> 10	8.6 (± 1.1)%
S3		> 10	9.5 (± 2.6)%
S10	Grade one (partial degradation)	> 10	35.6 (± 0.77)%
S14		> 10	57.2 (± 0.55)%
S2	Grade two (complete degradation)	~ 9.8	~ 100% (by projection)
S13		9	100%
S1		8–9	100%
S8		7–8	100%
S6		7	100%
S16		7	100%
S11		6–7	100%
S7		5	100%

Samples were assigned to one of three grades (zero, one and two) according to the completeness of dye decolorization, as well as the percent decolorization, at the end of the experiment

### Microbiome analysis

High-throughput sequencing of the DNA extracted from the 16 stool samples generated 2,579,071 reads (mean = 161,012.5 reads per sample). Quality assessment and paired-read-joining resulted in 2,520,799 filtered reads (mean reads per sample = 157,670.50), which were used for analysis. Rarefaction curves confirmed a reasonable coverage, sufficient to analyze the dominant members of the bacterial communities and to compare between samples, and all samples were rarefied to the smallest observed number of reads (121,509).

### Microbiome profile and gut microbiome biomarker ratios

**Fig. 1:**
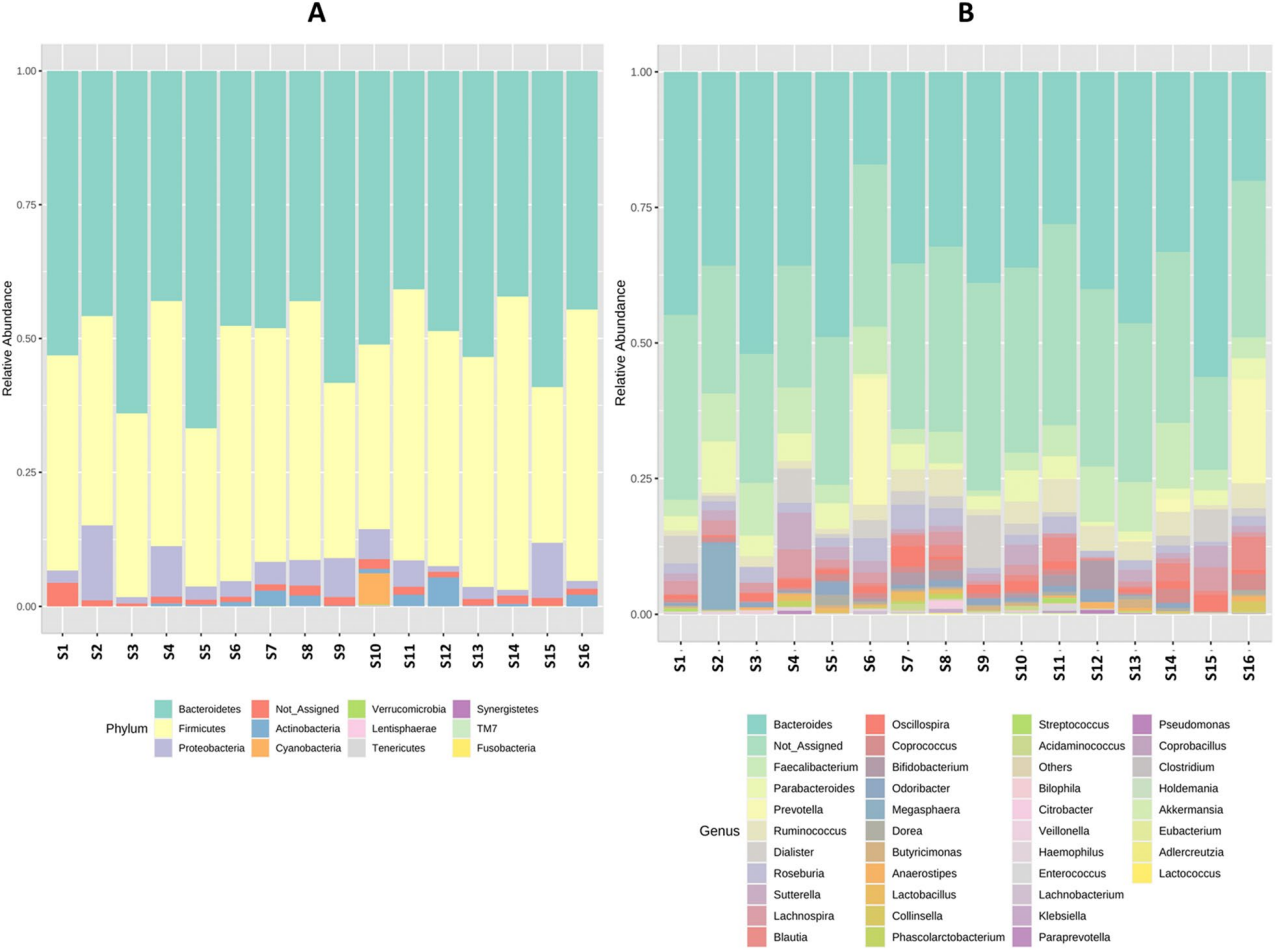
Taxonomic summary of the microbial communities detected in the fecal samples at the phylum level (**A**) and genus level (**B**). Only the 40 genera with the highest mean relative abundance, across all samples, are shown

At the phylum level, 11 different phyla were identified, four of which were the most predominant in all samples: Bacteroidetes, with relative abundance ranging from 40.3 to 66.1% (mean = 50.19%), followed by Firmicutes (relative abundance range: 29.2 to 54.7%, mean = 41.65%), Proteobacteria (relative abundance range: 1.1 to 14.1%, mean = 4.87%), and Actinobacteria (relative abundance range: 0 to 5.4%, mean = 1.12%). Seven other phyla were found in low proportions, while unidentified bacterial sequences ranged in relative abundance from 0.005 to 0.053% (Fig. 1A).

At the genus level, 174 genera were observed, 36 of which (representing 88.13% of the total microbial community) were shared by all samples, and were thus considered “core genera”. Seventeen genera (representing 0.007% of the entire community) were unique to one sample each (known as singleton genera), while 121 other genera, present in some but not all samples (non-core genera) represented 3.95% of the entire community (Fig. 1B).

Commonly used gut microbiome biomarkers were estimated. The Firmicutes-to-Bacteroidetes ratio ranged from 0.45 to 1.3 (mean = 0.87); the *Prevotella*-to-*Bacteroides* ratio ranged from 0.000067 to 1.36 (mean = 0.15); and the *Fusobacterium*-to-*Bifidobacterium* ratio, which was only measurable in six samples that had detectable *Fusobacterium* sequences ranged from 0.001 to 0.5 (Table 2).

**Table 2:** Selected gut microbiome biomarkers among analyzed fecal samples

Sample number	Azo-reducing activity grade	Firmicutes-to-Bacteroidetes ratio	*Prevotella*-to-*Bacteroides* ratio	*Fusobacterium*-to-*Bifidobacterium* ratio
S1	Two	0.778	0.000067	0
S2	Two	0.867	0.005	0
S3	Zero	0.532	0.0005	0
S4	Zero	1.064	0.0009	0
S5	Zero	0.453	0.003	0
S6	Two	0.987	1.36	0.004
S7	Two	0.918	0.0002	0.001
S8	Two	1.124	0.002	0
S9	Zero	0.696	0.0002	0.002
S10	One	0.553	0.0005	0.048
S11	Two	1.254	0.009	0
S12	Zero	0.903	0.0002	0
S13	Two	1.304	0.072	0
S14	One	0.81	0.007	0.4
S15	Zero	0.494	0.00099	0.5
S16	Two	1.133	0.965	0

*Prevotella*-to-*Bacteroides* ratio > 1 is double-underlined and *Fusobacterium*-to-*Bifidobacterium* ratios > 0 are underlined

### Compositional variations in relative abundance of key taxa and gut microbiome biomarkers in relation to azo-reducing activity

**Fig. 2:**
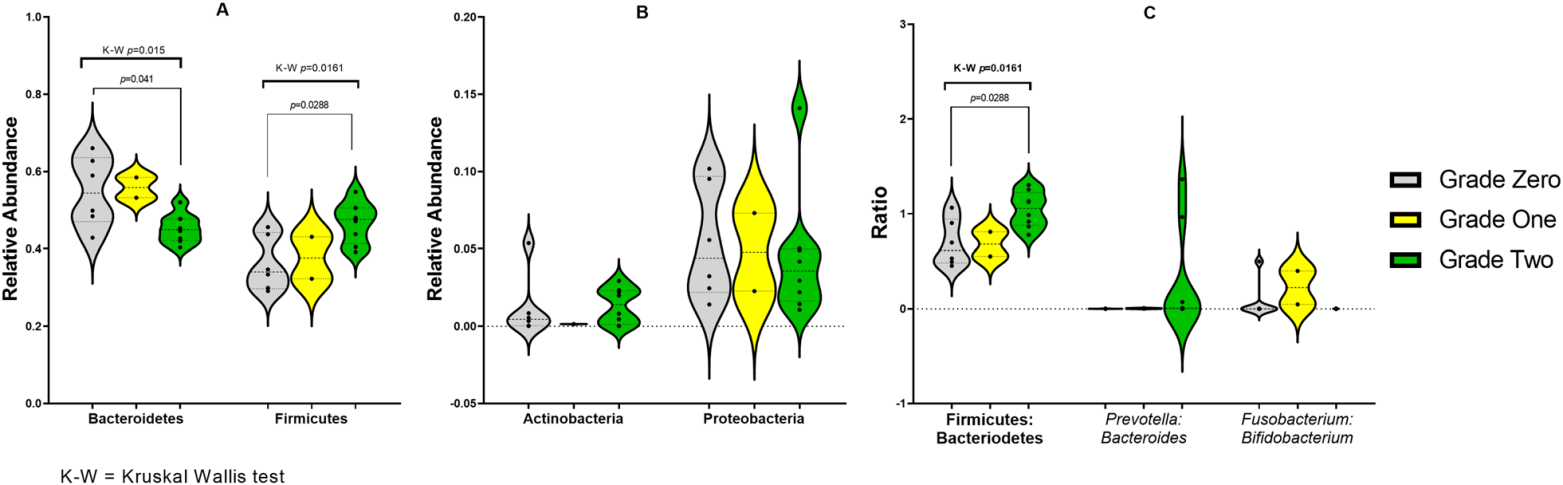
Bean/Violin plots representing the relative abundance of core phyla (**A** and **B**) and gut microbiome biomarkers (**C**) among fecal samples of different grades of azo-reducing activity. Phyla in panels **A** and **B** are separated because of different scales. *p-*values of significant differences (*p* < 0.05), as assessed by Kruskal–Wallis test, are indicated above each plot. *p-*values of significant differences upon pairwise post hoc tests are indicated as well

According to the extent of their azo-reducing activity (or lack thereof), the different samples significantly varied in the relative abundance of both Bacteroidetes and Firmicutes (Kruskal–Wallis test *p*-value < 0.05, Fig. 2A). As both Firmicutes and Bacteroidetes relative abundances independently had a significant, but reciprocal, impact on the observed azoreductase activity, the Firmicutes-to-Bacteroidetes ratio was expectedly significantly different between grades (Kruskal–Wallis test *p*-value = 0.0161, Fig. 2C).

In addition, the *Prevotella*-to-*Bacteroides* ratio considerably varied among samples, with the lowest median within ‘grade zero’ samples (ratio = 0.00072 or 1:1394), and relatively higher ratios in ‘grade one’ (0.00388 or 1:258) and ‘grade two’ (0.00706 or 1:142). Despite these striking differences (Additional file 1: Figure S2), the results did not reach statistical significance owing to ‘grade-two’ samples’ bimodal distribution (Fig. 2C): while the samples with relatively high *Prevotella*-to-*Bacteroides* ratio were among high-degraders, not all high-degraders had high ratios.

Hierarchical clustering of the samples’ composition at the phylum level highlights the above-mentioned inverse relation in the relative abundance of Bacteroidetes and Firmicutes (Additional file 1: Figure S3).

The significant variation in relative abundance of Bacteroidetes and Firmicutes among different grades of azo-reducing activity were tracked to lower taxonomic levels. At the family level, only family Bacteroidaceae, out of eight families under order Bacteroidales, significantly varied in relative abundance among the three grades (Kruskal–Wallis test *p*-value = 0.0106).

**Fig. 3:**
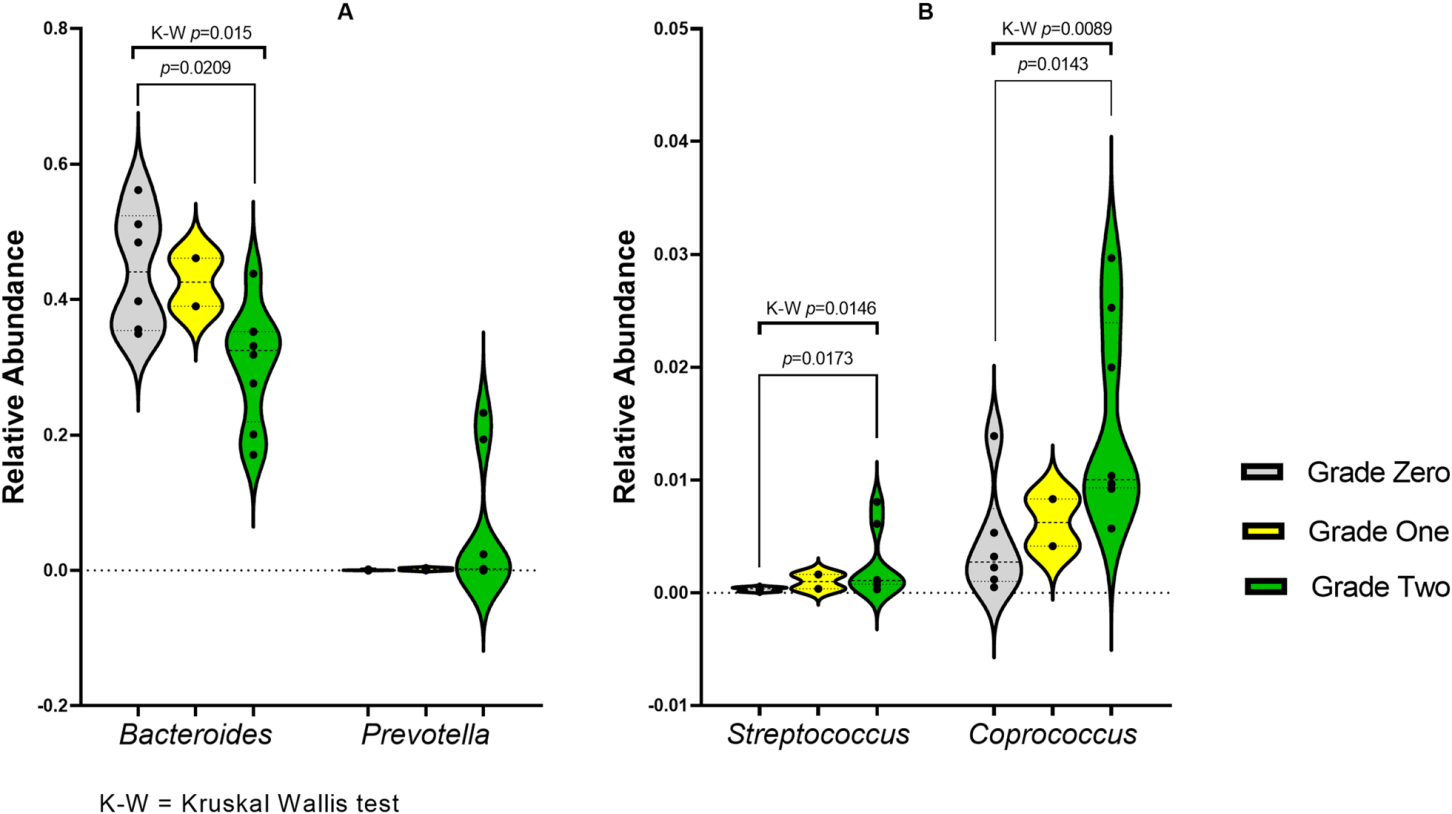
Bean/Violin plots representing the relative abundance of different genera of phyla Bacteroidetes (**A**) and Firmicutes (**B**) among groups of different azo-reducing activity grade. *p-*values of significant differences (*p* < 0.05), as assessed by Kruskal–Wallis test, as well as post hoc pairwise tests are indicated above each plot

The relative abundance of genus *Bacteroides*, the only representative of Family Bacteroidaceae, was significantly different (Kruskal–Wallis test *p*-value = 0.015) among the three grades (Fig. 3A), with its highest relative abundance among non-degrading samples (median = 0.4409).

Among the different families of phylum Firmicutes, only family Streptococcaceae was significantly variable among the three groups (Kruskal–Wallis test *p*-value = 0.0127). Finally, at the genus level, genus *Streptococcus* of family Streptococcaceae and genus *Coprococcus* of family Lachnospiraceae significantly varied in their relative abundance among groups (Kruskal–Wallis test *p*-value = 0.0146 and 0.0089, respectively, Fig. 3B).

Although their relative abundance was not significantly different among groups at the phylum level (Fig. 2B), Actinobacteria and Proteobacteria had some families and genera with significantly different relative abundance among the three grades: these are the actinobacterial family Corynebacteriaceae (essentially genus *Corynebacterium*, Kruskal–Wallis test *p*-value = 0.0302), and the proteobacterial genera *Lautropia* (family Burkholderiaceae) and *Paracoccus* (family Rhodobacteraceae)—both with Kruskal–Wallis test *p*-value = 0.0302.

**Fig. 4:**
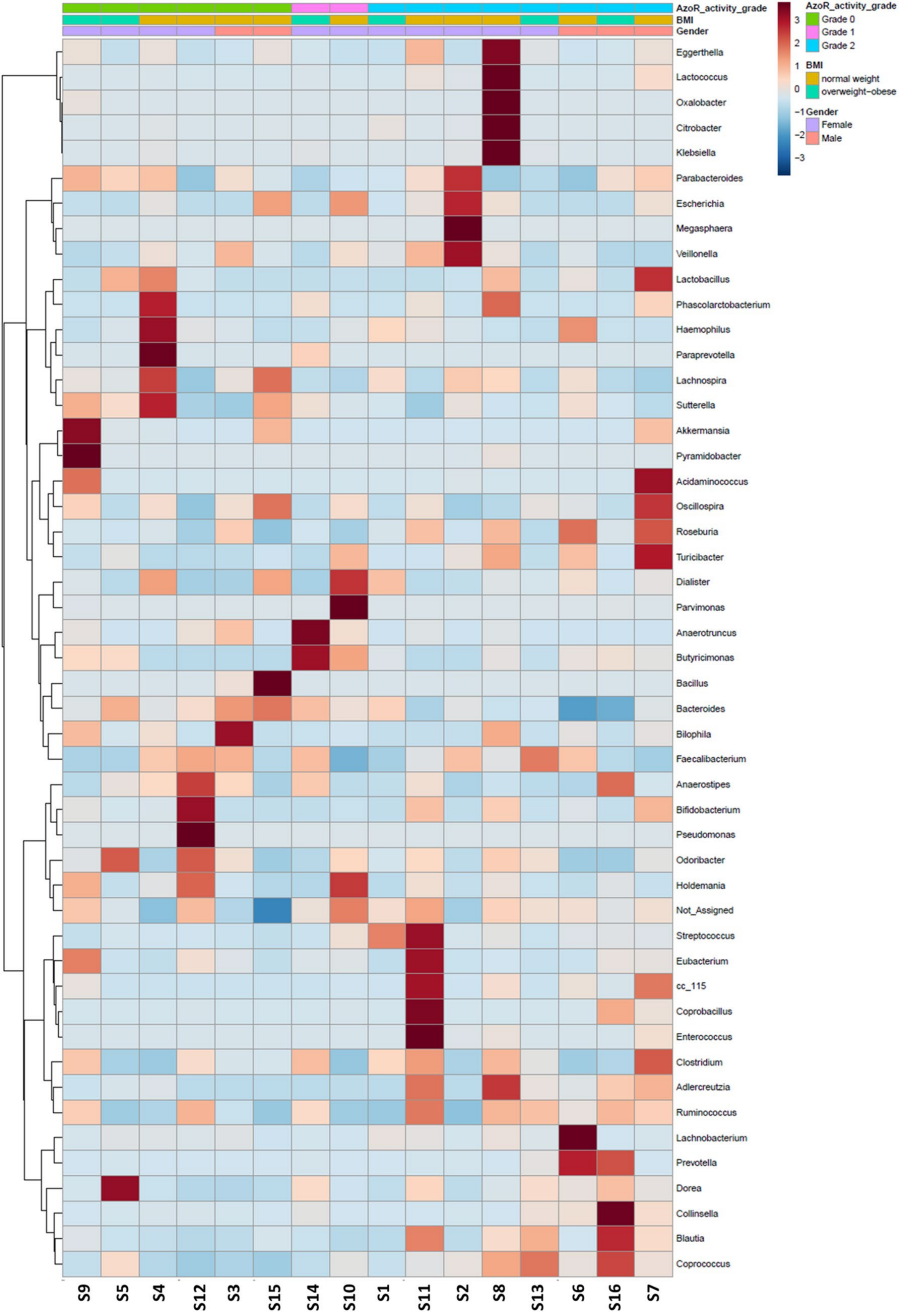
Heatmap visualization of hierarchical clustering of the gut microbiota composition at the genus level. Samples are categorized according to different criteria and arranged according to their azo-reducing activity. Colors on top of the heatmap represent the azo-reducing activity to which samples belong, subject sex, and subject BMI category. Heatmap color (blue to dark red) displays the row-scaled relative abundance of each taxon across all samples. Clustering was based on Euclidean distances

Hierarchical clustering of the samples’ composition at the genus level points out to higher relative abundance of genera *Coprococcus*, *Ruminococcus*, *Blautia* and *Adlercreutzia* in samples of detectable azo-reducing activity (grade-one and grade-two samples). On the other hand, genus *Bacteroides* was more abundant in non-degrading samples (Fig. 4).

### Alpha and beta diversity analyses

**Fig. 5:**
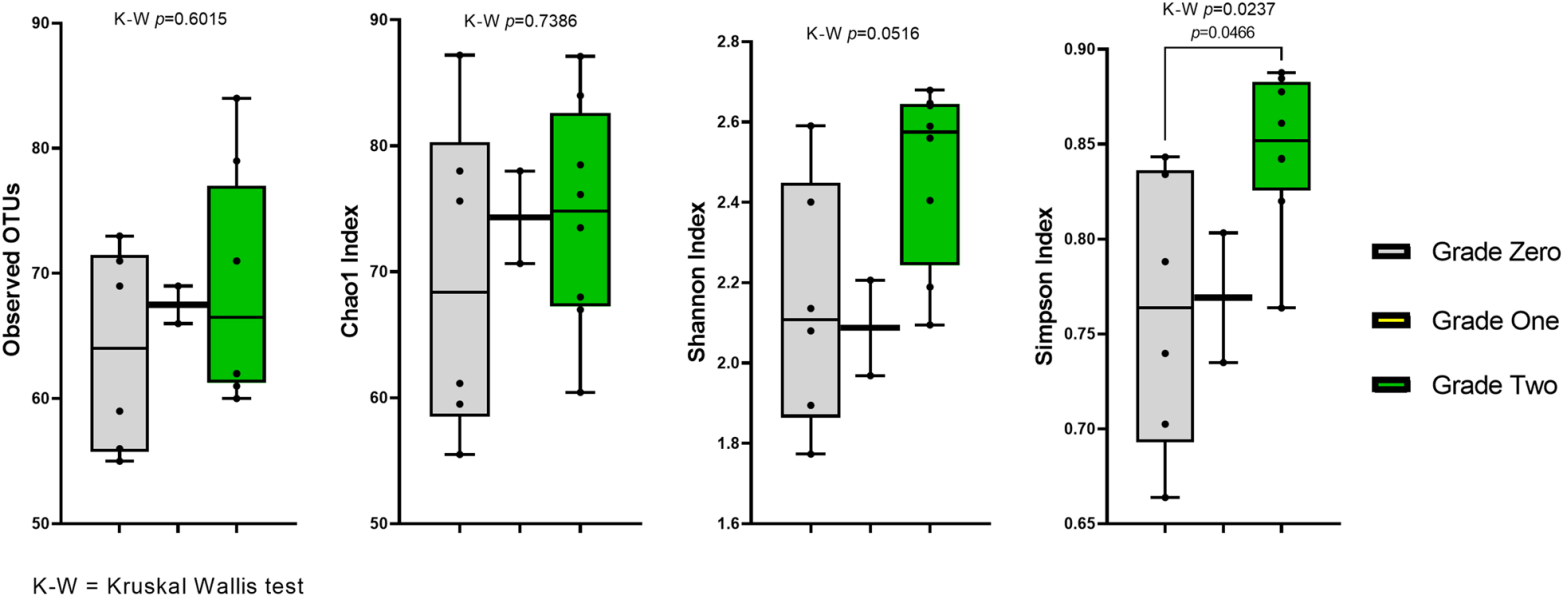
Boxplots representing different alpha diversity metrics (observed OTUs, Chao1, Shannon, and Simpson diversity indices) among different azo-reducing activity grades. Significant differences were estimated by Kruskal–Wallis test, followed by non-parametric pairwise post hoc tests, and *p*-values that are < 0.05 are shown

None of the common alpha diversity metrics was significant different between sexes or BMI groups (Additional file 1: Figures S4 and S5), while the azo-reducing activity grade of the samples significantly affected their diversity, but not richness (Fig. 5). On the other hand, analysis of diversity between sample groups (beta diversity) did not show any clear clustering pattern.

### Multivariate analysis

Because of the small sample size, and because of BMI variations among the study subjects, we performed multivariate analysis using linear models to estimate the extent by which each of the measured variables contributes to diversity metrics and relative abundance differences between taxa. Specifically, we sought to verify whether the changes seen among Bacteroides, Firmicutes, and alpha diversity are truly associated with the azo-reducing activity of the samples or are rather due to confounding factors, such as BMI (Additional file 1: Table S1). Azo-reducing activity was significantly associated with the relative abundance of Firmicutes and Bacteroidetes (*p*-values = 0.0081 and 0.0007, respectively). Bacteroidetes relative abundance was additionally significantly affected by sex and BMI (*p*-values = 0.0087 and 0.0451, respectively). On the other hand, both age and BMI were significantly associated with Actinobacteria abundance (*p*-value = 0.0148 and 0.0215, respectively, Additional file 1: Table S1).

Likewise, a multivariate analysis with the same four covariates vs*.* gut biomarker ratios singled out the Firmicutes-to-Bacteroidetes ratio as a significant covariate with azo-reducing activity (*p*-value = 0.0045, Additional file 1: Table S2).

Multivariate analysis of azo-reducing activity, age, sex, and BMI vs. alpha diversity indices indicated no significant contribution of azo-reducing activity, sex, or BMI to sample richness, while age was only a significant covariate with Chao1 index of richness. Meanwhile, multivariate analysis confirmed that Simpson diversity index was a predictor of azo-reducing grade (*p*-value = 0.0329, Additional file 1: Table S3).

## Discussion

The human gut contains trillions of metabolically active microbial cells that enrich the human gene pool with millions of genes, and their encoded enzymes. Azoreductases (expressed by several members of the human gut microbiota) greatly affect metabolism of azodyes, extensively used in food and pharmaceuticals. Thus, the gut microbiota composition is expected to affect the metabolism of many drugs and azodye-containing compounds, and administering these azodye-containing drugs/xenobiotics to different populations, without taking their gut microbiota composition in consideration, might affect the metabolism and bioavailability of such drugs.

In this study, the core microbiome of 16 stool samples, collected from the same neighborhood to reduce variations based on diet and lifestyle, was defined by 16S rRNA amplicon sequencing. This analysis, with an admittedly small sample size, is intended to be a pilot comparative analysis of microbiome structure to relate gut microbial communities to their overall azo-reducing activity. In spite of the deluge of microbiome studies in the past few years, only a handful gut microbiome studies were conducted in Egypt (e.g., [22–26]), and none of them focused on xenobiotic-degrading phenotypes.

Early microbiome studies reported that Firmicutes and Bacteroidetes dominated in the gut (~ 90% relative abundance), but to highly variable degrees [27, 28], and suggested the Firmicutes-to-Bacteroidetes ratio as a significant a biomarker for the human gut microbiota status [29], as the coexistence of Bacteroidetes and Firmicutes in the gut implies minimized competition for resources [30]. Another important biomarker of the gut microbiota status/health is the *Prevotella*-to-*Bacteroides* ratio, which was suggested as a predictor for successful body fat loss, notably on diets high in fiber and whole grain [31, 32].

In our study, the microbiome profile of the fecal samples had a typical gut microbiome signature, as Firmicutes and Bacteroidetes constituted ~ 92% of microbial populations. A key finding of the study is that high azo-reducing activity positively correlated with phylum Firmicutes but negatively with phylum Bacteroidetes. This might be because the genomes of Firmicutes are rich in azoreductase-encoding genes [4]. However, Proteobacteria supersede Firmicutes as azoreductase producers; yet their relative abundance did not significantly or consistently vary within different stool samples, which might be due to their lower overall relative abundance in the gut microbiota in comparison to Firmicutes and Bacteroidetes. Consequently, high azo-reducing stool samples had a higher Firmicutes-to-Bacteroidetes median ratio than low- or non-degraders.

In addition, the median *Prevotella*-to-*Bacteroides* ratio was higher in the high azo-reducing group, but did not reach statistical significance owing to high within-group variability. Although some *Bacteroides* species are known for their azo-reducing activity [33, 34], while no azoreductases have been described in *Prevotella* species, *Prevotella* might be relatively enriched in some high azo-reducing samples just because of its inverse correlation with *Bacteroides*, whereas the actual activity was due to the members of phylum Frimicutes in such samples. It is often the case that when *Bacteroides* is high in a sample, *Prevotella* is low, and vice versa [35]. Another interpretation of the high variability in *Prevotella* relative abundance among azo-reducers, might be that an azoreductase activity is yet to be discovered in some *Prevotella* species, or that the activity is strain specific, and thus cannot be resolved by 16S rRNA analysis.

At the genus level, significant variation in alpha diversity was observed with the Simpson diversity index, while richness was not significantly different. This result suggests that evenness, rather than number of taxa is what differentiates the groups. Beta diversity of samples classified according to their azo-reducing activity (and estimated by the weighted UniFrac method) indicated no particular clustering patterns. A possible interpretation is that, although Firmicutes seemed to clearly have an effect on the final azoreductase activity, different genera of Firmicutes were dominant in different samples, and no particular clustering of taxa was observed; yet, the presence of any of these genera seemed to encode enough azoreductases.

The *Fusobacterium*-to-*Bifidobacterium* ratio is considered as a biomarker for dysbiosis of the gut microbiota. Patients with colorectal cancer were reported to have a decrease in the relative abundance of *Bifidobacterium* coupled with increases in *Faecalibacterium prausnitzii* abundance [36, 37]. Unsurprisingly, in this study, *Faecalibacterium* species was nil in 10 samples and of negligible value in the other six samples, as all our samples were collected from subjects with no reported diseases (other than obesity in one subject).

Finally, we performed multivariate analysis of relative abundances of different bacterial taxa and gut microbiome biomarkers with age, sex, and BMI of participants to rule out that the observed associations were caused by a confounding factor. Invariably, azo-reducing activity was found as a key player in the relative abundance of Firmicutes and one of the significant covariates with Bacteroidetes relative abundance, and consequently the Firmicutes-to-Bacteroidetes ratio. BMI, on the other hand, was a key covariate with Bacteroidetes and Actinobacteria. These results are in accordance with Kim et al.’s report that phylum Actinobacteria was positively associated with body weight [38]. In addition an investigation of gut microbiota of lean and obese twins observed higher levels of Actinobacteria in obese subjects [39]. Kim et al. also reported that age significantly increased the proportions of both class Coriobacteriia and family Coriobacteriaceae in phylum Actinobacteria [38], whereas La-ongkham et al. observed that the relative abundance of the phylum Actinobacteria in the adult subjects was significantly higher by approximately 2.3 times than that in the elderly group [40]. Here, age was found as a significant covariate with Chao 1 richness and with Actinobacteria relative abundance, but not with that of the three other major phyla.

## Conclusion

In conclusion, we analyzed the fecal microbiomes of 16 adult Egyptian volunteers in a pilot study to relate composition of microbial communities to their azo-reducing activity. Major taxa usually associated with the human gut environment were observed, indicating a typical gut microbiome signature. Despite the small sample size, using multivariate followed by univariate analyses indicated statistically significant trends. The microbiome profiling indicated variations in the Firmicutes-to-Bacteroides and *Prevotella*-to-*Bacteroides* ratios among samples with different azo-reducing grades, suggesting the relative abundance of phylum Firmicutes as the most striking factor that may have affected the final azo-reducing activity. Additionally, samples with different azo-reducing grades significantly differed in evenness.

The major limitations of this work are the small sample size and the absence of evidence of causality behind observed statistical associations. Future studies should address these limitations by analyzing larger cohorts; by combining and comparing samples from different human populations at different geographical locations, representing different diets and lifestyles; and by using animal models or ex vivo models of the gut microbiota to allow investigating causality (e.g., by experimentally altering the Firmicutes-to-Bacteroidetes ratio and measuring the azoreductase activity). Additionally, shotgun metagenomics, metaproteomics, metabonomics, and functional metagenomics strategies (reviewed and compared in [12]) are all likely to provide insight into the mechanism of microbiome members involvement in the azoreduction process.

Moreover, the results of this study highlight the importance of characterizing azoreductase-producing gut bacteria, notably among *Bacteroides* and *Prevotella* species, which have not been as studied as Firmicutes and Proteobacteria, and which may have important strain-level variations. Such studies will help increase our knowledge about the fate of azodye-containing drugs or chemicals, and about differential human responses to them. These results will also guide the development of more efficient drugs and dosage forms, and will contribute to efforts for implementing microbiome testing in precision medicine and toxicology.

## Methods

### Study subjects and sample collection

Sixteen volunteers, from which azo-reducing bacteria were previously isolated [7], were the source of stool samples analyzed in this study, and their metadata were recorded (Table 3). The subjects had no chronic or infectious diseases, no previous history of gastrointestinal disease, and had not been prescribed antibiotics for at least 3 months prior to specimen collection. The specimens were stored at − 20 °C for further DNA extraction; but for detection of total azodye decolorization activity, samples were used while fresh, before they were frozen.

**Table 3:** Metadata of volunteers from whom stool samples were collected

Sample number	Age	Gender	Approximate weight (kg)	Approximate height (m)	Body Mass Index (BMI)	BMI category^a^
S1	26	Female	70	1.65	25.71	Overweight
S2	20	Female	60	1.65	22.04	Normal
S3	20	Male	58	1.55	24.14	Normal
S4	24	Female	60	1.66	21.77	Normal
S5	40	Female	75	1.5	33.33	Obese
S6	33	Male	83	1.83	24.78	Normal
S7	25	Male	70	1.7	24.22	Normal
S8	24	Female	60	1.55	24.97	Normal
S9	29	Female	67	1.63	25.22	Overweight
S10	20	Female	54	1.66	19.6	Normal
S11	22	Female	55	1.7	19.03	Normal
S12	38	Female	60	1.6	23.44	Normal
S13	35	Female	60	1.47	27.77	Overweight
S14	29	Female	75	1.62	28.58	Overweight
S15	20	Male	49	1.52	21.21	Normal
S16	40	Male	72	1.6	28.13	Overweight

^a^Normal weight range: 18.5–24.9, overweight range: 25–29.9, and obese of 30 or greater

### Total decolorization activity of stool samples

The total Brilliant Black-decolorization activity of a given stool sample was determined, in 50 ml brain heart infusion-supplemented (BHIS) broth containing 50 µl of 0.06 M Brilliant Black solution, by the method described by McConnell and Tannock [41]. The percent decolorization was estimated in triplicates for each sample. The concentration of the azodye was determined from a standard curve for calibration of known concentrations of Brilliant Black. Samples were classified into three grades: grade zero or non-degraders, grade one or partial degraders, and grade two or complete degraders.

### DNA extraction

The QIAamp^®^ DNA Stool Mini Kit (Qiagen, Germany) was used for DNA extraction. The manufacturer’s instructions were followed exactly. DNA was initially quantified in a NanoDrop spectrophotometer, and prior to sequencing in a Qubit fluorometer (Thermo Fisher Scientific, Waltham, MA, USA).

### 16S rRNA amplicon sequencing

The yield and purity of DNA were checked in a Nanophotometer^®^ P-330 (Implen, Germany) to ensure its suitability for sequencing. DNA was sequenced at Centros FISABIO, Valencia, Spain with an Illumina Miseq™ Sequencer as per the manufacturer’s instructions. Paired-ends (2 × 300 bp) protocol was performed with the universal primers (341F 5′-CCTACGGGNGGCWGCAG-3′ and 805R 5′-GACTACHVGGGTATCTAATCC-3) covering V3–V4 16S rRNA gene regions [42]. The amplicon library was generated by the Illumina amplicon library protocol (Part #15044223 Rev. A), with Illumina Nextera indexes (Illumina, San Diego, CA, USA). The 16S rRNA gene amplicons and subsequent index PCR products were purified with AgenCourt AMPure XP beads (Beckman Coulter, Indianapolis, IN, USA).

### Bioinformatics analysis of 16S rRNA sequence data

Quality assessment, as well as cleaning and trimming were performed by FastQC [43] and PrinSeq [44] respectively, to give out two cleaned fastq files for each sample. Cleaned forward and reverse reads were combined in a single contig to give a joined-reads fastq file for each sample. Joined sequences were analyzed in QIIME software version 1.9 [45]. OTU picking, taxonomic identification and phylogenetic alignment were performed by the “pick_open_reference_otus.py” script based on 97% identity with the Greengenes database version 13.8 [46], and any reads which do not hit the reference sequence collection are subsequently clustered de novo. “core_diversity_analyses.py” script was then performed to calculate alpha and beta diversity using different metrics. The QIIME analysis pipeline uses ‘usearch’ for chimera detection and low abundance cluster filtering. QIIME output and taxonomic analysis data are provided (Additional files 2 and 3, respectively).

### Statistical analyses

Several statistical tests were automatically performed as part of QIIME pipeline, and were confirmed by GraphPad Prism version 9.0 software and MicrobiomeAnalyst web-based tool for comprehensive statistical, visual and meta-analysis of microbiome data [47]. The tests used included: multivariate analysis with linear models, ANOVA or Kruskal–Wallis tests for parametric and non-parametric data, respectively, and Student’s t-test or Mann–Whitney test for parametric and non-parametric comparisons between two variables, respectively).

## Supplementary Information

**Additional file 1.** Supplementary figures (Figures S1-S5) and tables (Tables S1-S3).

**Additional file 2.** QIIME output in Biological Observation Matrix (biom) data format for allsamples.

**Additional file 3.** An Excel file with multiple sheets, representing relative abundance ofdifferent taxa (phyla, classes, order, family, genera).

## Data Availability

All sequences were deposited in NCBI under Project accession # PRJNA702535 (SRA Experiments: SRX10121839 to SRX10121854), and the samples were assigned the Serial numbers: SAMN17974787 to SAMN17974802. Processed and analyzed data are provided in two additional files (Additional files 2 and 3, respectively).
