# Supplementary material for: Compositional variation of the human fecal microbiome in relation to azo-reducing activity: a pilot study
Source: Gut Pathog. 2021 Oct 8;13:58. doi: 10.1186/s13099-021-00454-0 (PMC8499468; doi:10.1186/s13099-021-00454-0)
Supplement: Supplementary file 1 — Additional file 1. Supplementary figures (Figures S1-S5) and tables (Tables S1-S3). [file 13099_2021_454_MOESM1_ESM.pdf]

Compositional variation of the human fecal microbiome in relation to azo-reducing activity: a pilot study

Sara A. Zahran[1], Marwa Ali-Tammam[1], Amal E. Ali[1], Ramy K. Aziz[2,3,4*]

[1]Department of Microbiology and Immunology, Faculty of Pharmacy, Future University in Egypt, 11835 New Cairo, Egypt
[2]Department of Microbiology and Immunology, Faculty of Pharmacy, Cairo University, 11562 Cairo, Egypt
[3]The Center for Genome and Microbiome Research, Faculty of Pharmacy, Cairo University, 11562 Cairo, Egypt
[4]Microbiology and Immunology Research Program, Children's Cancer Hospital Egypt 57357, 11617 Cairo, Egypt
* Correspondence: **E-mail:** ramy.aziz@gmail.com

SUPPLEMENTARY MATERIAL

Content:

Additional file 1 (this file):

Figure S1: Total azoreduction by stool samples.

Figure S2: Log-scaled boxplots of the *Prevotella*-to-*Bacteroides* ratio among the three grades of azo-reduction.

Figure S3: Heatmap visualization of hierarchical clustering of the gut microbiota composition at the phylum level.

Figure S4: Boxplots comparing alpha diversity metrics in males and females.

Figure S5: Boxplots comparing alpha diversity metrics among different BMI groups.

Supplementary Tables: Multivariate analysis with partial-least squares (PLS) regression of different covariates *vs.* key phyla relative abundance (S1), gut microbiome biomarker ratios (S2), and alpha diversity metrics (S3).

Additional data files:

- **Additional file 2:** QIIME output in Biological Observation Matrix (biom) data format for all samples
- **Additional file 3:** An Excel file with multiple sheets, representing relative abundance of different taxa (phyla, classes, order, family, genera)

Figure S1:

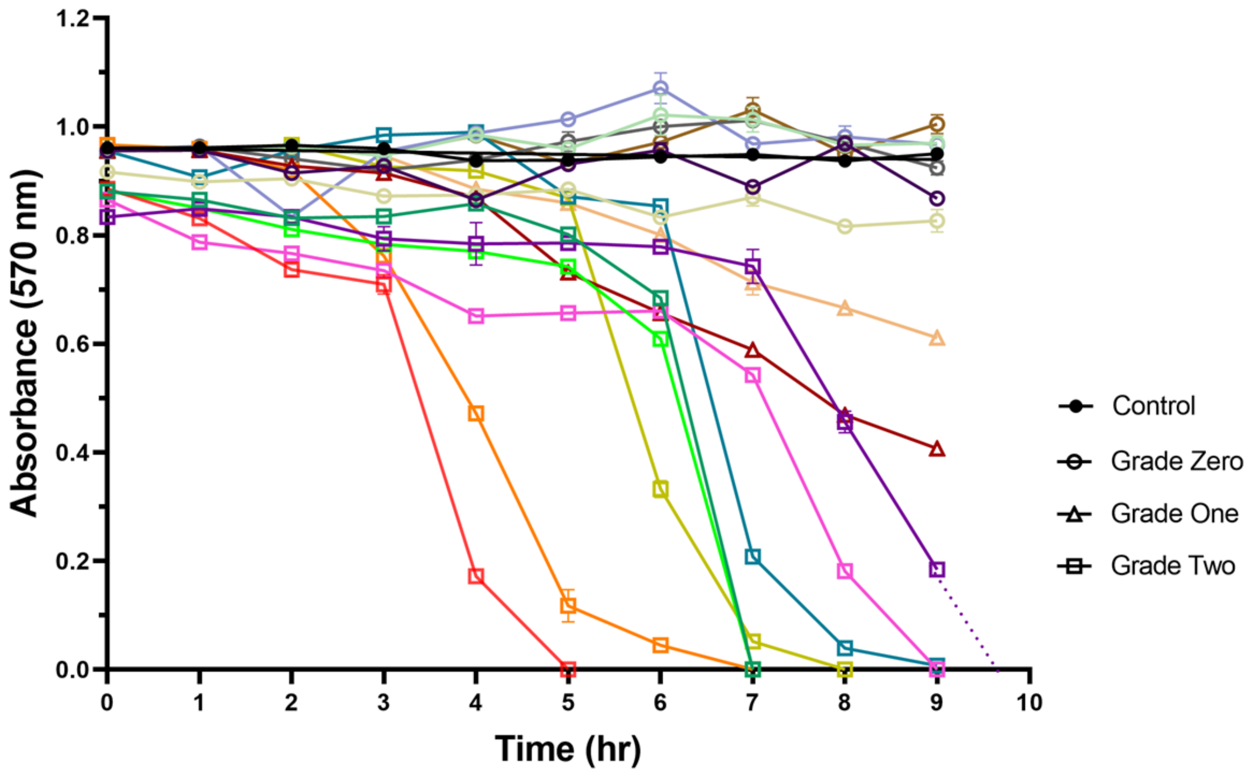

Total azoreduction by stool samples. Line plots representing the decrease in absorbance *vs.* time by different stool samples incubated with 0.06M Brilliant Black for 10 hours. Absorbance was measured hourly. Different samples (n = 16) are shown in different colors, and the corresponding negative control (blank) is shown in a black line with solid circles. Different azo-reduction grades are shown in different shapes (open circles = grade zero; open triangles = grade one; open squares = grade two).

<u>Figure S2</u>:

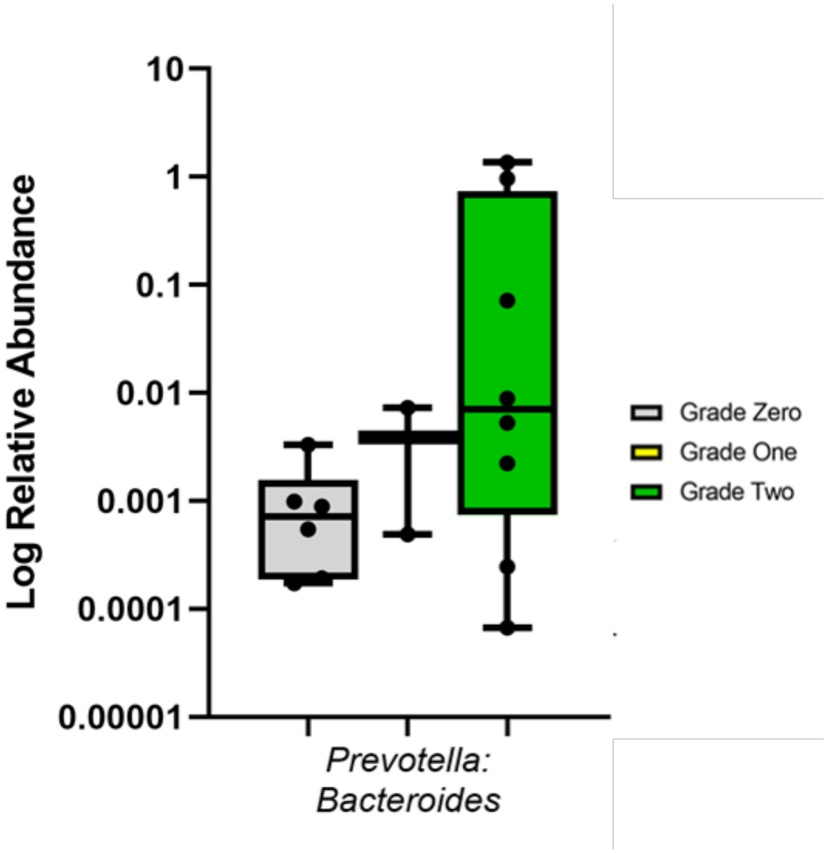

Boxplots of the *Prevotella*-to-*Bacteroides* ratio among the three groups of samples, based on their azo-reducing activity, represented on a log-scale (Log 10) to emphasize the difference between low ratios (see Figure 2C for a linear scale, in context of other variables).

<u>Figure S3</u>:

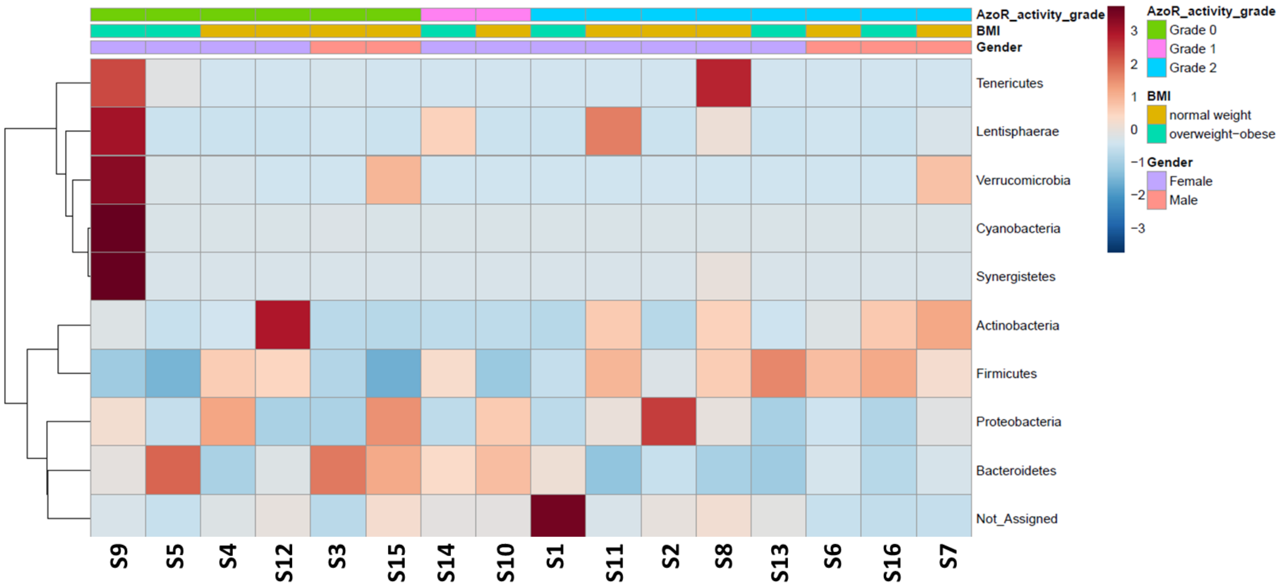

Heatmap visualization of hierarchical clustering of the gut microbiota composition at the phylum level. Samples are categorized according to different criteria and arranged according to their azo-reducing activity. Colors on top of the heatmap represent the azo-reducing activity to which samples belong, subject sex and subject BMI category. Heatmap color (blue to dark red) displays the row-scaled relative abundance of each taxon across all samples. Clustering was based on Euclidean distances. The figure was generated by *MicrobiomeAnalyst* (URL: https://www.microbiomeanalyst.ca)

Figure S4:

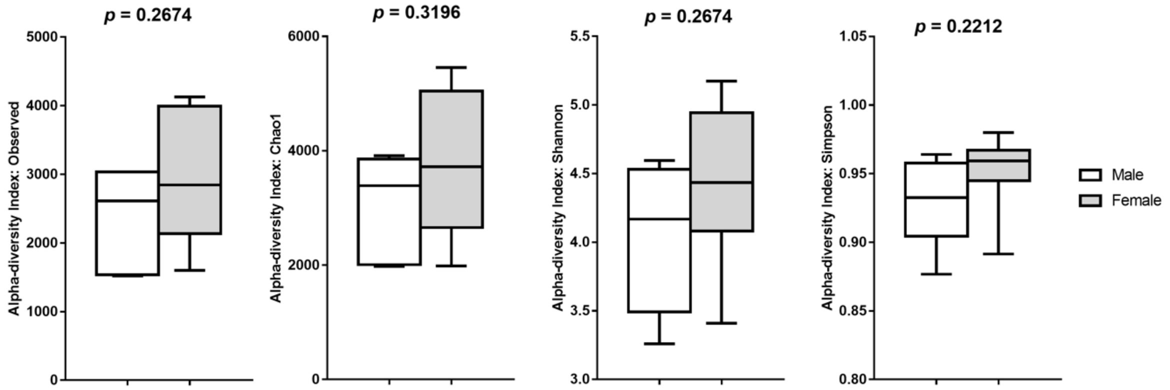

Boxplots comparing alpha diversity metrics in males and females. Alpha diversity metrics (observed OTUs, Chao1, Shannon and Simpson indices) are compared between both sexes. No significant differences were observed (Mann Whitney test *p*-value > 0.05).

Figure S5:

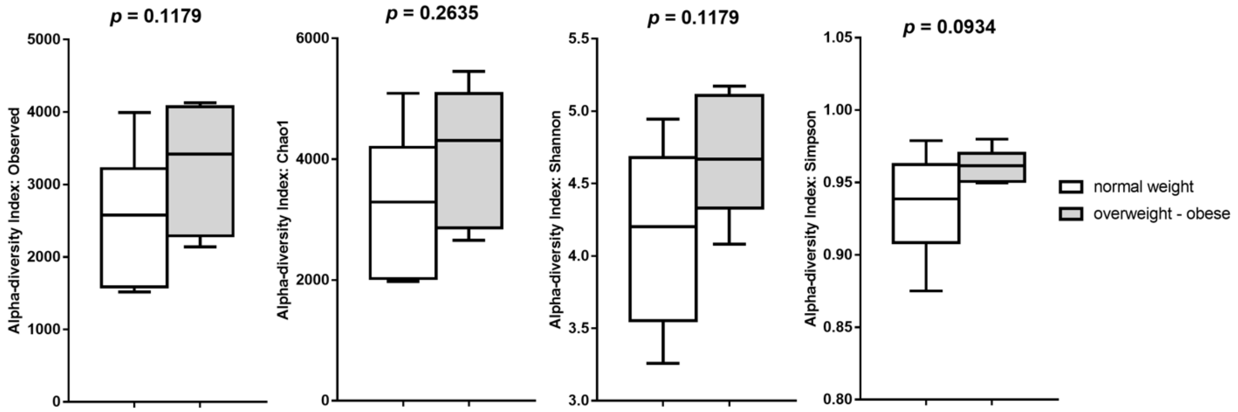

Boxplots comparing alpha diversity metrics among BMI groups. Observed OTUs, Chao1, Shannon and Simpson indices are compared in different BMI groups. No significant differences were observed. Mann Whitney test *p*-values are shown above each plot.

<u>Table S1</u>: Multivariate analysis with partial least squares (PLS) regression of composite variables *vs.* the **relative abundance of the main gut phyla**: Actinobacteria, Bacteroidetes, Firmicutes, and Proteobacteria

<table>
<tr><th rowspan="2">Independent Variables</th><th colspan="3">Dependent variable: Firmicutes</th></tr>
<tr><td>ANOVA p-value</td><td>Regression p-value</td><td>R squared</td></tr>
<tr><td>Azo-reducing activity</td><td>0.0081*</td><td rowspan="4">0.0252*</td><td rowspan="4">0.6786</td></tr>
<tr><td>Gender</td><td>0.1968</td></tr>
<tr><td>BMI</td><td>0.4319</td></tr>
<tr><td>Age</td><td>0.1423</td></tr>
<tr><th rowspan="2">Independent Variables</th><th colspan="3">Dependent variable: Bacteroidetes</th></tr>
<tr><td>ANOVA p-value</td><td>Regression p-value</td><td>R squared</td></tr>
<tr><td>Azo-reducing activity</td><td>0.0007***</td><td rowspan="4">0.0016**</td><td rowspan="4">0.8221</td></tr>
<tr><td>Gender</td><td>0.0087**</td></tr>
<tr><td>BMI</td><td>0.0451*</td></tr>
<tr><td>Age</td><td>0.0592</td></tr>
<tr><th rowspan="2">Independent Variables</th><th colspan="3">Dependent variable: Actinobacteria</th></tr>
<tr><td>ANOVA p-value</td><td>Regression p-value</td><td>R squared</td></tr>
<tr><td>Azo-reducing activity</td><td>0.8399</td><td rowspan="4">0.1289</td><td rowspan="4">0.5294</td></tr>
<tr><td>Gender</td><td>0.507</td></tr>
<tr><td>BMI</td><td>0.0215*</td></tr>
<tr><td>Age</td><td>0.0148*</td></tr>
<tr><th rowspan="2">Independent Variables</th><th colspan="3">Dependent variable: Proteobacteria</th></tr>
<tr><td>ANOVA p-value</td><td>Regression p-value</td><td>R squared</td></tr>
<tr><td>Azo-reducing activity</td><td>0.8722</td><td rowspan="4">0.316</td><td rowspan="4">0.4052</td></tr>
<tr><td>Gender</td><td>0.4841</td></tr>
<tr><td>BMI</td><td>0.3507</td></tr>
<tr><td>Age</td><td>0.2452</td></tr>
</table>

* $p < 0.05$, ** $p < 0.01$, *** $p < 0.001$

<u>**Table S2**</u>**:** Multivariate analysis with partial least squares (PLS) regression of composite variables *vs.* various **gut microbiome biomarkers** (Firmicutes-to-Bacteroidetes, *Prevotella*-to-*Bacteroides* and *Fusobacterium*-to-*Bifidobacterium* ratios).

**Independent Variables**	**Dependent variable: Firmicutes-to-Bacteroidetes**		
	ANOVA *p*-value	Regression *p*-value	R squared
Azo-reducing activity	0.0045**	0.0148*	0.7146
Gender	0.0768		
BMI	0.2514		
Age	0.1507		
**Independent Variables**	**Dependent variable: *Prevotella*-to-*Bacteroides***		
	ANOVA *p*-value	Regression *p*-value	R squared
Azo-reducing activity	0.3915	0.2286	0.4553
Gender	0.0507#		
BMI	0.8499		
Age	0.5666		
**Independent Variables**	**Dependent variable: *Fusobacterium*-to-*Bifidobacterium***		
	ANOVA *p*-value	Regression *p*-value	R squared
Azo-reducing activity	0.2026	0.2723	0.4292
Gender	0.3171		
BMI	0.7898		
Age	0.3402		

* p< 0.05, ** p<0.01 & # marginal significance

<u>Table S3</u>: Multivariate analysis with partial least squares (PLS) regression of composite variables *vs.* **alpha diversity indices** at the genus level (Observed OTUs, Chao1, Shannon, and Simpson).

**Independent Variables**	**Dependent variable: Observed OTUs**		
	ANOVA *p*-value	Regression *p*-value	R squared
Azo-reducing activity	0.3674	0.1335	0.5253
Gender	0.1381		
BMI	0.6843		
Age	0.0949		
**Independent Variables**	**Dependent variable: Chao1 Index**		
	ANOVA *p*-value	Regression *p*-value	R squared
Azo-reducing activity	0.5729	0.1772	0.4902
Gender	0.4099		
BMI	0.8183		
Age	0.0414*		
**Independent Variables**	**Dependent variable: Shannon Index**		
	ANOVA *p*-value	Regression *p*-value	R squared
Azo-reducing activity	0.086	0.2686	0.4313
Gender	0.5341		
BMI	0.4843		
Age	0.3601		
**Independent Variables**	**Dependent variable: Simpson Index**		
	ANOVA *p*-value	Regression *p*-value	R squared
Azo-reducing activity	0.0329*	0.0978	0.56
Gender	0.2985		
BMI	0.2127		
Age	0.1493		

* $p < 0.05$
